# Lipidomics dataset of sonication-induced traumatic optic neuropathy in mice

**DOI:** 10.1016/j.dib.2020.105147

**Published:** 2020-01-16

**Authors:** Ronaldo Nuesi, Ryan A. Gallo, Galina Dvoriantchikova, Daniel Pelaez, Sanjoy K. Bhattacharya

**Affiliations:** Bascom Palmer Eye Institute, Department of Ophthalmology, University of Miami, Miami, FL, 33136, USA

**Keywords:** Traumatic optic neuropathy, Optic nerve injury, Lipid profile, Liquid chromatography-mass spectrometry, Metabolomics, Neurodegeneration

## Abstract

Traumatic optic neuropathy (TON) is the loss of vision secondary to trauma. Approximately two weeks after traumatic damage, diffuse retinal ganglion cell loss and axon degeneration of the optic nerve are exhibited [1]. Here we present the changes that occur in the optic nerve lipidome of two-month-old C57BL/6J mice following sonication-induced TON (SI-TON), which closely models the indirect clinical mechanism in TON. Optic nerves were harvested at three time points following injury: 1-day, 7-days, and 14-days for comparison with the control group (uninjured optic nerves from 2-month-old mice). The optic nerves were subjected to mass spectrometry and bioinformatic analysis using LipidSearch 4.1.3 and Metaboanalyst 4.0. This data pertains to the lipidome at each time point following indirect trauma to the optic nerve. The data presented here will augment investigation into the neurodegenerative process. The data is available at Metabolomics Workbench [http://www.metabolomicsworkbench.org (Project ID: PR000859)].

**Table:** Specifications Table

Subject	Cell Biology
Specific subject area	Lipids, cell membranes
Type of data	Table Figure
How data were acquired	Liquid Chromatography Q-Exactive Orbitrap Mass Spectrometry, LipidSearch 4.1.3, Metaboanalyst 4.0
Data format	Raw Analyzed Filtered
Parameters for data collection	Optic nerve, age, survival
Description of data collection	Optic nerves were dissected beginning at the optic nerve head and ending right before the optic chiasm, Methyl-Tert-Butyl Ether lipid extraction was performed, and lipids were analyzed with LC-MS/MS.
Data source location	Bascom Palmer Eye Institute, Miller School of Medicine at University of Miami, Miami, FL 33136, USA
Data accessibility	Repository name: Metabolomics Workbench- Project ID: PR000859 Data identification number: https://doi.org/10.21228/M8NH5V Direct URL to data: https://www.metabolomicsworkbench.org/data/DRCCMetadata.php?Mode=Project&ProjectID=PR000859
Related research article	Tao, W. et al., *A Novel Mouse Model of Traumatic Optic Neuropathy Using External Ultrasound Energy to Achieve Focal, Indirect Optic Nerve Injury.* Sci Rep, 2017.**7** (1): p. 11779.

**Table:** 

**Value of the Data** • The data depicts changes in the optic nerve lipidome at post sonication injury time points compared to control pertinent to retinal ganglion cell loss (demonstrated previously) providing insight into lipid differences at the cellular level during injury induced axon degeneration. • The data can be used to examine other optic neuropathies and broader neurodegeneration by investigators interested in changes at the cellular level following traumatic injuries. • The data can serve as a template for specific lipid classes and species to assess behaviour of these lipids in the neurodegenerative process, for multi-omics studies and for providing information on these lipids that can be used to facilitate further experimentation utilizing specific species. • This data will specifically serve as baseline changes in lipids in traumatic optic neuropathy model (induced using sonic wave) for pharmacological and biologics treatment. This data will also serve as potential lipidomics baseline for other traumatic injuries to optic nerve for comparative studies.

## 1 Data description

**Fig. 1:**
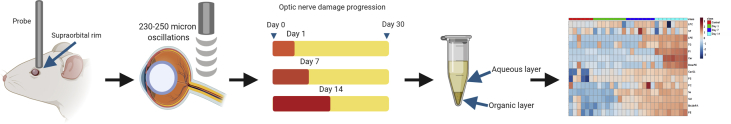
**Schematic diagram of C57BL/6J mice exhibiting lipidomic changes following ultrasonicated ocular trauma.** A probe was placed on the supraorbital rim and 500msec pulses were transmitted with 60–80 J of force. Mice optic nerves were harvested at one day, seven days, and fourteen days after sonic wave exposure. MTBE extraction was performed and lipids collected from the upper organic layer. Lipid changes in the optic nerve were analyzed using mass spectrometry and a heat map was generated.

**Fig. 2:**
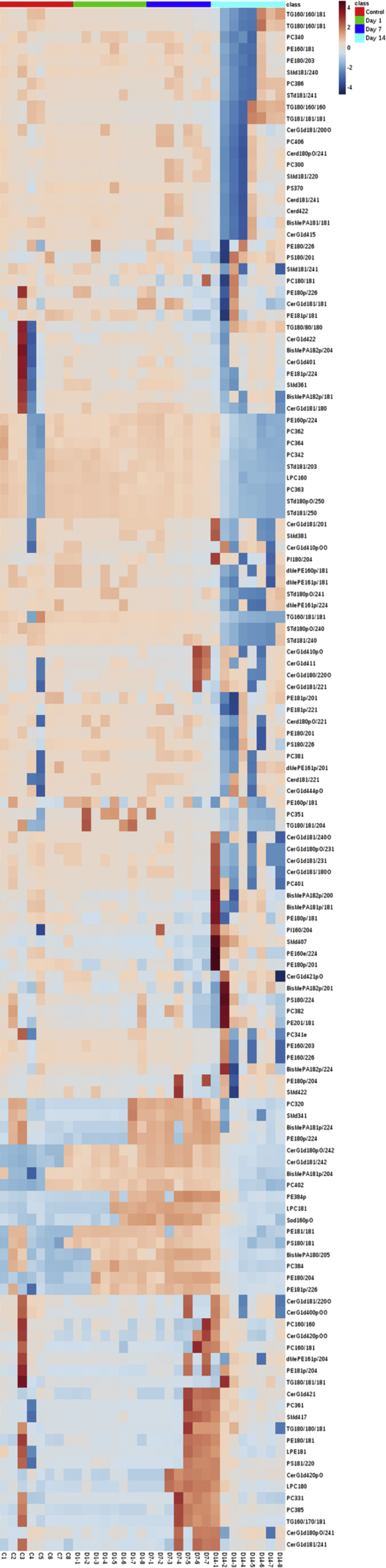
**Lipidome heatmap.** Relative abundance of lipid species with control (no exposure) and 1-day, 7-days and 14-days post exposure to sonication. All outliers were included and 56 significant species were identified by One Way ANOVA. (p-value set to 0.05).

Here we present a lipid profiling of the optic nerve following sonication-induced trauma in two-month-old C57BL/6J mice and that from the control mice. Mice were placed in a sound-proof chamber and exposed to a 500msec sonic shock from a microtip probe as shown in Fig. 1. Optic nerve samples were collected at 1 day, 7 days, and 14 days post sonication and subjected to Methyl-Tert-Butyl Ether/Methanol (MTBE) lipid extraction. BCA Assay was used to aliquot lipids corresponding to a 30μg by protein in each sample. Lipids were re-suspended in Chloroform: Methanol (1:1) and processed through untargeted liquid chromatography Q-Exactive Orbitrap tandem mass spectrometry (LC-MS/MS). Relative quantification was performed using lipid peaks of the species identified with LipidSearch 4.1.3 software. Data from LipidSearch 4.1.3 was formatted and exported to Metaboanalyst 4.0 for statistical analyses as shown in Fig. 2. Labeling of samples were as in Table 1. A list of lipid nomenclature used can be found in Table 2 and identified lipids can be found in Supplementary Table S1.

**Table 1:** Sample Identification. 31 optic nerve samples were used from 15 males and 16 females. Each sample was run twice in positive mode and twice in negative mode.

Sample	Exposure	Days Post Exposure	Sex	ESI Mode
C1	No Sonication	0	M	Pos
C1	No Sonication	0	M	Neg
C2	No Sonication	0	M	Pos
C2	No Sonication	0	M	Neg
C3	No Sonication	0	M	Pos
C3	No Sonication	0	M	Neg
C4	No Sonication	0	M	Pos
C4	No Sonication	0	M	Neg
C5	No Sonication	0	F	Pos
C5	No Sonication	0	F	Neg
C6	No Sonication	0	F	Pos
C6	No Sonication	0	F	Neg
C7	No Sonication	0	F	Pos
C7	No Sonication	0	F	Neg
C8	No Sonication	0	F	Pos
C8	No Sonication	0	F	Neg
D1_1	Sonication	1	M	Pos
D1_1	Sonication	1	M	Neg
D1_2	Sonication	1	M	Pos
D1_2	Sonication	1	M	Neg
D1_3	Sonication	1	M	Pos
D1_3	Sonication	1	M	Neg
D1_4	Sonication	1	M	Pos
D1_4	Sonication	1	M	Neg
D1_5	Sonication	1	F	Pos
D1_5	Sonication	1	F	Neg
D1_6	Sonication	1	F	Pos
D1_6	Sonication	1	F	Neg
D1_7	Sonication	1	F	Pos
D1_7	Sonication	1	F	Neg
D1_8	Sonication	1	F	Pos
D1_8	Sonication	1	F	Neg
D7_1	Sonication	7	F	Pos
D7_1	Sonication	7	F	Neg
D7_2	Sonication	7	F	Pos
D7_2	Sonication	7	F	Neg
D7_3	Sonication	7	F	Pos
D7_3	Sonication	7	F	Neg
D7_4	Sonication	7	F	Pos
D7_4	Sonication	7	F	Neg
D7_5	Sonication	7	M	Pos
D7_5	Sonication	7	M	Neg
D7_6	Sonication	7	M	Pos
D7_6	Sonication	7	M	Neg
D7_7	Sonication	7	M	Pos
D7_7	Sonication	7	M	Neg
D14_1	Sonication	14	M	Pos
D14_1	Sonication	14	M	Neg
D14_2	Sonication	14	M	Pos
D14_2	Sonication	14	M	Neg
D14_3	Sonication	14	M	Pos
D14_3	Sonication	14	M	Neg
D14_4	Sonication	14	M	Pos
D14_4	Sonication	14	M	Neg
D14_5	Sonication	14	F	Pos
D14_5	Sonication	14	F	Neg
D14_6	Sonication	14	F	Pos
D14_6	Sonication	14	F	Neg
D14_7	Sonication	14	F	Pos
D14_7	Sonication	14	F	Neg
D14_8	Sonication	14	F	Pos
D14_8	Sonication	14	F	Neg

**Table 2:** LipidSearch nomenclature.

Group	Abbreviations	Lipid Name
P-Choline	LPC	lysophosphatidylcholine
	PAF	platelet-activating factor
	PC	phosphatidylcholine
	MePC	Methyl phosphatidylcholine

P-Ethanol Amine	LPE	lysophosphatidylethanolamine
	LdMePE	lysodimethylphosphatidylethanolamine
	PE	phosphatidylethanolamine
	BisMePE	Bis-methyl phosphatidylethanolamine
	dMePE	dimethylphosphatidylethanolamine

P-Serine	LPS	lysophosphatidylserine
	PS	phosphatidylserine
	BisMePS	Bis-methyl phosphatidy lserine

P-Glycerol	LPG	lysophosphatidylglycerol
	PG	phosphatidylglycerol
	BisMePG	Bis-methyl phosphatidylglycerol

P-Inositol	LPI	lysophosphatidylinositol
	PI	phosphatidylinositol
	PIP	phosphatidylinositol
	PIP2	phosphatidylinositol
	PIP3	phosphatidylinositol

P-Ethanol	LPEt	lysophosphatidylethanol
	PEt	phosphatidylethanol

P-Acid	LPA	lysophosphatidic acid
	BisMeLPA	Bis-methyl lysophosphatidic acid
	PA	phosphatidic acid
	BisMePA	Bis-methyl phosphatidic acid
	cPA	cyclic phosphatidic acid

P-Methanol	LPMe	lysophosphatidylmethanol
	PMe	phosphatidylmethanol

Sphingolipids	SM	sphingomyelin
	LSM	lysosphingomyelin
	phSM	sphingomyelin (phytosphingosine)

Neutral glycerolipid	MG	monoglyceride
	DG	diglyceride
	TG	triglyceride

Fatty Acid	FA	fatty acid

Cardiolipin	CL	Cardiolipin

Sphingoid base	So	Sphingosine
	SoP	Sphingosine phosphate

Neutral Glycosphingolipids	SoG1	Glucosylsphingosine
	CerG1	Simple Glc series
	CerG2	Simple Glc series
	CerG3	Simple Glc series
	CerG2GNAc1	Simple Glc series
	CerG3GNAc1	Simple Glc series
	CerG3GNAc2	Simple Glc series
	ST	Sulfatide

Glycosphingolipids	Cer	Ceramides
	CerP	Ceramides phosphate
	GM3	Gangliosides
	GM2	Gangliosides
	GM1	Gangliosides
	GD1a	Gangliosides
	GD1b	Gangliosides
	GD2	Gangliosides
	GD3	Gangliosides
	GT1a	Gangliosides
	GT1b	Gangliosides
	GT1c	Gangliosides
	GT2	Gangliosides
	GT3	Gangliosides
	GQ1c	Gangliosides
	GQ1b	Gangliosides

Steroid	ChE	Cholesterol Ester
	ZyE	zymosterol
	StE	Stigmasterol ester
	SiE	Sitosterol ester
	AGlcSiE	AcylGlcSitosterol ester
	D7ChE	Deuterated Cholesterol Ester

Coenzyme	Co	Coenzyme

Fatty Ester	OAHFA	(O-acyl)-1-hydroxy fatty acid
	WE	wax exters
	AcCa	Acyl Carnitine

Glycoglycerolipid	MGMG	Monogalactosylmonoacylglycerol
	MGDG	Monogalactosyldiacylglycerol
	DGMG	Digalactosylmonoacylglycerol
	DGDG	Digalactosyldiacylglycerol
	SQMG	Sulfoquinovosylmonoacylglycerol
	SQDG	Sulfoquinovosyldiacylglycerol

Neutral glycerolipid (deuterated)	D5DG	Deuterated diglyceride
	D5TG	Deuterated triglyceride

## 2 Experimental design, materials, and methods

### 2.1 Animals

All animals were treated in accordance with the Association for Research in Vision and Ophthalmology (ARVO) statement for the use of animals in ophthalmic and vision research, and were used under protocols approved by the University of Miami, Institutional Animal Care and Use Committee (IACUC). C57BL/6J mice were obtained from Jackson Laboratory (Bar Harbor, ME, USA). Mice were maintained in a temperature-regulated environment with a 12-h light, 12-h dark cycle, and all mice were fed ad libitum. Two-month-old mice were used for this dataset.

### 2.2 Sonication-induced traumatic optic neuropathy model

Sonication-induced traumatic optic neuropathy (SI-TON) model was performed as described previously [1]. Briefly, TON was induced in two-month-old C57BL/6J mice with a Branson Digital Sonifier 450 (Branson Ultrasonics, Danbury, CT, USA) by a 3mm microtip probe in an acoustic soundproof enclosure chamber. Mice were anesthetized with vaporized isoflurane supplied with oxygen in an induction chamber. The fur adjacent to each mouse's supraorbital rim was shaved, and each mouse was placed on the stage of a sound-proof enclosure equipped with an anesthesia mask for continuous supply of anesthesia. The stage was adjusted so that the microtip probe was in direct contact with the supraorbital rim above the insertion point of the optic nerve into the optic canal. Only left optic nerves were injured. The sonicator was programmed to deliver a 500 msec shock at a 35% or 40% amplitude, which results in a 230 to 250-μm oscillation according to the manufacturers' specifications. After sonication, mice were placed in a new cage with thermal support until fully recovered.

### 2.3 MTBE lipid extraction

Optic nerves were carefully dissected at 1 day, 7 days, and 14 days post exposure, beginning from the optic nerve head and continuing on until reaching the optic chiasm. Methyl-*tert*-butyl ether (MTBE) extraction was then performed as described with some modifications [2]. Briefly, optic nerves were immersed in 400 μl of Methanol + BHT then snap frozen and thawed for five-minute cycles using liquid nitrogen and a 37° water bath until completely homogenized. Samples were transferred to amber glass vials and 1.3 mL of MTBE was added. They were incubated in the dark at 4 °C on an orbital shaker overnight. The following day, samples were transferred to a centrifuge tube and 417 μl of 0.15 M Ammonium Acetate was added. They were then centrifuged for 10 min at 2000×*g* at 4 °C. The organic layer (upper) was collected, transferred to 2mL glass vials and dried in a centrifugal vacuum concentrator. Samples were re-suspended in 50 μl of 1:1 Chloroform: Methanol and stored in −20 °C until further processing.

### 2.4 High performance liquid chromatography and mass spectrometry

Lipids were analyzed by liquid chromatography electrospray tandem mass spectrometry (LC-MS/MS) using an Accela HPLC system and an orbitrap mass spectrometer (Q Exactive, Thermo Scientific, Waltham MA). An Acclaim 120 C18 3μm column (Thermo Scientific) was used with LC-MS grade Methanol: Water 60:40 v/v with 10mM Ammonium Acetate and Methanol Chloroform 60:40 v/v with 10mM Ammonium Acetate, as solvent A and B, respectively. A Heated Electrospray Ionization Source (HESI) was operated at a spray voltage of 4.4kV, a HESI vaporization temperature of 275 °C, a sheath gas pressure of 45 arbitrary units, and an auxillary gas flow of 15 arbitrary units. The ion transfer tube was kept at a temperature of 350 °C. The scan range was set at 150–1500 *m*/*z*. The gradient ran at 35%–100% Solvent B for 13 minutes and then was held at 35% solvent B for 2 minutes. The gradient was then brought up to 100% solvent A for 3 minutes and held for 2 minutes.

### 2.5 Lipid identification and relative quantification

Raw data from LC-MS was uploaded to LipidSearch 4.1.3 (Thermo Scientific). The search parameters were as follows: productsearch, precursor (5/5) ppm, intensity threshold 1.0%, M-Score 0.0. Quantitation and Toprank filter were turned on, Main node filters were set to Main Isomer Peaks, and ID quality was graded from A-D. All target classes were selected with the exception of fatty esters, glycoglycerolipids, and deuterated glycerolipids. All adducts in negative mode were selected, and all adducts in positive mode were selected with the exceptions of Li+, (CH3CH2)3NH +, and (CH3) 2NH2 +.

### 2.6 Data analysis

After peaks were identified, every sample was aligned to calculate the unassigned peaks. During alignment, lipid identification was filtered by grading from A-C. A few peaks in the following lipid classes of CerG1, PC, LPC, PE, TG, and ST were rejected as false positives and removed. Data was placed into four groups (Control, One day, Seven Days, Fourteen Days) and statistical analysis was performed with Metaboanalyst 4.0. No missing values were detected. Data was normalized to the reference group (control), log transformation was applied, and heat maps were then generated (Fig. 2).
