# Supplementary material for: Lipidomics dataset of sonication-induced traumatic optic neuropathy in mice
Source: Data Brief. 2020 Jan 16;29:105147. doi: 10.1016/j.dib.2020.105147 (PMC6994518; doi:10.1016/j.dib.2020.105147)
Supplement: Multimedia component 1 [file mmc1.pdf]

Supplementary Table S1

Lipid Class	Lipid Species	Calculated Mass	Formula	Base Retention Time
BisMePA	BisMePA(18:0/20:5)	750.52	C43 H75 O8 N0 P1	12.587
	BisMePA(18:0/20:5)	750.52	C43 H75 O8 N0 P1	13.327
	BisMePA(18:1/18:1)	728.5356	C41 H77 O8 N0 P1	13.82
	BisMePA(18:1p/18:1)	712.5407	C41 H77 O7 N0 P1	13.874
	BisMePA(18:1p/20:4)	734.525	C43 H75 O7 N0 P1	13.376
	BisMePA(18:1p/22:4)	762.5563	C45 H79 O7 N0 P1	14.126
	BisMePA(18:2p/18:1)	710.525	C41 H75 O7 N0 P1	13.012
	BisMePA(18:2p/20:0)	740.572	C43 H81 O7 N0 P1	14.674
	BisMePA(18:2p/20:1)	738.5563	C43 H79 O7 N0 P1	14.006
	BisMePA(18:2p/20:4)	732.5094	C43 H73 O7 N0 P1	12.505
	BisMePA(18:2p/22:4)	760.5407	C45 H77 O7 N0 P1	13.343
Cer	Cer(d18:0+pO/22:1)	637.6009	C40 H79 O4 N1	13.19
	Cer(d18:0+pO/24:1)	665.6322	C42 H83 O4 N1	14.271
	Cer(d18:1/22:1)	619.5903	C40 H77 O3 N1	13.196
	Cer(d18:1/24:1)	647.6216	C42 H81 O3 N1	14.277
	Cer(d42:2)	647.6216	C42 H81 O3 N1	13.666
CerG1	CerG1(d18:0+pO/23:1)	813.6694	C47 H91 O9 N1	13.75
	CerG1(d18:0+pO/24:1)	827.685	C48 H93 O9 N1	14.257
	CerG1(d18:0+pO/24:2)	825.6694	C48 H91 O9 N1	13.269
	CerG1(d18:0/22:0+O)	801.6694	C46 H91 O9 N1	13.576
	CerG1(d18:1/18:0)	727.5962	C42 H81 O8 N1	11.255
	CerG1(d18:1/18:0+O)	743.5911	C42 H81 O9 N1	10.73
	CerG1(d18:1/18:1)	725.5806	C42 H79 O8 N1	10.761
	CerG1(d18:1/20:0+O)	771.6224	C44 H85 O9 N1	12.008
	CerG1(d18:1/20:1)	753.6119	C44 H83 O8 N1	12.009
	CerG1(d18:1/22:0+O)	799.6537	C46 H89 O9 N1	13.192
	CerG1(d18:1/22:1)	781.6432	C46 H87 O8 N1	13.196
	CerG1(d18:1/23:1)	795.6588	C47 H89 O8 N1	13.75
	CerG1(d18:1/24:0+O)	827.685	C48 H93 O9 N1	14.973
	CerG1(d18:1/24:1)	809.6745	C48 H91 O8 N1	14.262
	CerG1(d18:1/24:1)	809.6745	C48 H91 O8 N1	14.522
	CerG1(d18:1/24:1)	809.6745	C48 H91 O8 N1	14.966
	CerG1(d18:1/24:2)	807.6588	C48 H89 O8 N1	13.279
	CerG1(d40:0+pO+O)	817.6643	C46 H91 O10 N1	13.189
	CerG1(d40:1)	783.6588	C46 H89 O8 N1	13.618
	CerG1(d41:0+pO)	815.685	C47 H93 O9 N1	14.166
	CerG1(d41:0+pO+O)	831.6799	C47 H93 O10 N1	13.745
	CerG1(d41:1)	797.6745	C47 H91 O8 N1	14.156
	CerG1(d41:5)	789.6119	C47 H83 O8 N1	13.786

	CerG1(d42:0+pO)	829.7007	C48 H95 O9 N1	14.576
	CerG1(d42:0+pO)	829.7007	C48 H95 O9 N1	15.219
	CerG1(d42:0+pO+O)	845.6956	C48 H95 O10 N1	14.27
	CerG1(d42:0+pO+O)	845.6956	C48 H95 O10 N1	14.968
	CerG1(d42:1)	811.6901	C48 H93 O8 N1	14.57
	CerG1(d42:1)	811.6901	C48 H93 O8 N1	15.216
	CerG1(d42:1+pO)	827.685	C48 H93 O9 N1	13.664
	CerG1(d42:2)	809.6745	C48 H91 O8 N1	13.667
	CerG1(d44:4+pO)	849.6694	C50 H91 O9 N1	13.661
LPC	LPC(16:0)	495.3325	C24 H50 O7 N1 P1	4.572
	LPC(16:0)	495.3325	C24 H50 O7 N1 P1	24.161
	LPC(18:0)	523.3638	C26 H54 O7 N1 P1	5.606
	LPC(18:1)	521.3481	C26 H52 O7 N1 P1	4.902
LPE	LPE(18:1)	479.3012	C23 H46 O7 N1 P1	23.894
PC	PC(16:0/16:0)	733.5622	C40 H80 O8 N1 P1	12.349
	PC(16:0/16:0)	733.5622	C40 H80 O8 N1 P1	32.791
	PC(16:0/18:1)	759.5778	C42 H82 O8 N1 P1	11.695
	PC(16:0/18:1)	759.5778	C42 H82 O8 N1 P1	12.643
	PC(16:0/18:1)	759.5778	C42 H82 O8 N1 P1	33.111
	PC(18:0/18:1)	787.6091	C44 H86 O8 N1 P1	13.776
	PC(18:0/18:1)	787.6091	C44 H86 O8 N1 P1	34.209
	PC(30:0)	705.5309	C38 H76 O8 N1 P1	11.16
	PC(32:0)	733.5622	C40 H80 O8 N1 P1	11.317
	PC(33:1)	745.5622	C41 H80 O8 N1 P1	10.971
	PC(34:0)	761.5935	C42 H84 O8 N1 P1	13.502
	PC(34:1e)	745.5985	C42 H84 O7 N1 P1	13.107
	PC(34:2)	757.5622	C42 H80 O8 N1 P1	10.908
	PC(35:1)	773.5935	C43 H84 O8 N1 P1	12.421
	PC(36:1)	787.6091	C44 H86 O8 N1 P1	13.121
	PC(36:2)	785.5935	C44 H84 O8 N1 P1	12.072
	PC(36:2)	785.5935	C44 H84 O8 N1 P1	12.368
	PC(36:2)	785.5935	C44 H84 O8 N1 P1	12.912
	PC(36:3)	783.5778	C44 H82 O8 N1 P1	11.495
	PC(36:4)	781.5622	C44 H80 O8 N1 P1	11.129
	PC(36:4)	781.5622	C44 H80 O8 N1 P1	12.165
	PC(38:1)	815.6404	C46 H90 O8 N1 P1	14.607
	PC(38:2)	813.6248	C46 H88 O8 N1 P1	13.913
	PC(38:4)	809.5935	C46 H84 O8 N1 P1	12.206
	PC(38:4)	809.5935	C46 H84 O8 N1 P1	12.541
	PC(38:4)	809.5935	C46 H84 O8 N1 P1	13.304
	PC(38:5)	807.5778	C46 H82 O8 N1 P1	11.496

	PC(38:5)	807.5778	C46 H82 O8 N1 P1	12.422
	PC(38:6)	805.5622	C46 H80 O8 N1 P1	12.247
	PC(40:1)	843.6717	C48 H94 O8 N1 P1	15.287
	PC(40:2)	841.6561	C48 H92 O8 N1 P1	14.7
	PC(40:6)	833.5935	C48 H84 O8 N1 P1	13.349
PE	PE(16:0/18:1)	717.5309	C39 H76 O8 N1 P1	12.706
	PE(16:0/20:3)	741.5309	C41 H76 O8 N1 P1	12.285
	PE(16:0/22:6)	763.5152	C43 H74 O8 N1 P1	12.292
	PE(16:0e/22:4)	753.5672	C43 H80 O7 N1 P1	13.443
	PE(16:0p/18:1)	701.5359	C39 H76 O7 N1 P1	12.763
	PE(16:0p/18:1)	701.5359	C39 H76 O7 N1 P1	33.207
	PE(16:0p/22:4)	751.5516	C43 H78 O7 N1 P1	13.088
	PE(18:0/18:1)	745.5622	C41 H80 O8 N1 P1	13.108
	PE(18:0/18:1)	745.5622	C41 H80 O8 N1 P1	13.82
	PE(18:0/18:1)	745.5622	C41 H80 O8 N1 P1	34.231
	PE(18:0/20:1)	773.5935	C43 H84 O8 N1 P1	14.628
	PE(18:0/20:3)	769.5622	C43 H80 O8 N1 P1	13.385
	PE(18:0/20:4)	767.5465	C43 H78 O8 N1 P1	13.341
	PE(18:0/20:4)	767.5465	C43 H78 O8 N1 P1	33.742
	PE(18:0/22:6)	791.5465	C45 H78 O8 N1 P1	13.408
	PE(18:0/22:6)	791.5465	C45 H78 O8 N1 P1	33.791
	PE(18:0p/18:1)	729.5672	C41 H80 O7 N1 P1	13.874
	PE(18:0p/18:1)	729.5672	C41 H80 O7 N1 P1	34.292
	PE(18:0p/20:1)	757.5985	C43 H84 O7 N1 P1	14.675
	PE(18:0p/20:1)	757.5985	C43 H84 O7 N1 P1	35.247
	PE(18:0p/20:4)	751.5516	C43 H78 O7 N1 P1	33.773
	PE(18:0p/22:4)	779.5829	C45 H82 O7 N1 P1	14.141
	PE(18:0p/22:4)	779.5829	C45 H82 O7 N1 P1	34.58
	PE(18:0p/22:6)	775.5516	C45 H78 O7 N1 P1	13.444
	PE(18:0p/22:6)	775.5516	C45 H78 O7 N1 P1	33.839
	PE(18:1/18:1)	743.5465	C41 H78 O8 N1 P1	12.468
	PE(18:1/18:1)	743.5465	C41 H78 O8 N1 P1	12.949
	PE(18:1p/18:1)	727.5516	C41 H78 O7 N1 P1	13.015
	PE(18:1p/18:1)	727.5516	C41 H78 O7 N1 P1	33.463
	PE(18:1p/20:1)	755.5829	C43 H82 O7 N1 P1	13.995
	PE(18:1p/20:1)	755.5829	C43 H82 O7 N1 P1	34.422
	PE(18:1p/20:4)	749.5359	C43 H76 O7 N1 P1	12.506
	PE(18:1p/20:4)	749.5359	C43 H76 O7 N1 P1	32.918
	PE(18:1p/22:1)	783.6142	C45 H86 O7 N1 P1	14.776
	PE(18:1p/22:4)	777.5672	C45 H80 O7 N1 P1	13.339
	PE(18:1p/22:6)	773.5359	C45 H76 O7 N1 P1	12.613
	PE(20:1/18:1)	771.5778	C43 H82 O8 N1 P1	13.924
	PE(38:4p)	751.5516	C43 H78 O7 N1 P1	33.497

PI	PI(16:0/20:4)	858.5258	C45 H79 O13 N0 P1	32.749
	PI(18:0/20:4)	886.5571	C47 H83 O13 N0 P1	13.523
	PI(18:0/20:4)	886.5571	C47 H83 O13 N0 P1	33.889
PS	PS(18:0/18:1)	789.552	C42 H80 O10 N1 P1	13.543
	PS(18:0/18:1)	789.552	C42 H80 O10 N1 P1	14.154
	PS(18:0/18:1)	789.552	C42 H80 O10 N1 P1	34.579
	PS(18:0/20:1)	817.5833	C44 H84 O10 N1 P1	14.902
	PS(18:0/20:1)	817.5833	C44 H84 O10 N1 P1	35.502
	PS(18:0/22:4)	839.5676	C46 H82 O10 N1 P1	14.424
	PS(18:0/22:6)	835.5363	C46 H78 O10 N1 P1	13.772
	PS(18:1/22:0)	845.6146	C46 H88 O10 N1 P1	15.565
	PS(37:0)	805.5833	C43 H84 O10 N1 P1	33.084
SM	SM(d18:1/22:0)	786.6615	C45 H91 O6 N2 P1	13.493
	SM(d18:1/24:0)	814.6928	C47 H95 O6 N2 P1	14.53
	SM(d18:1/24:1)	812.6771	C47 H93 O6 N2 P1	13.529
	SM(d34:1)	702.5676	C39 H79 O6 N2 P1	7.763
	SM(d34:1)	702.5676	C39 H79 O6 N2 P1	9.777
	SM(d36:1)	730.5989	C41 H83 O6 N2 P1	11.025
	SM(d38:1)	758.6302	C43 H87 O6 N2 P1	12.276
	SM(d40:7)	774.5676	C45 H79 O6 N2 P1	14.583
	SM(d41:7)	788.5832	C46 H81 O6 N2 P1	13.197
	SM(d42:2)	812.6771	C47 H93 O6 N2 P1	33.989
ST	ST(d18:0+pO/24:0)	909.6575	C48 H95 O12 N1 S1	15.215
	ST(d18:0+pO/24:1)	907.6419	C48 H93 O12 N1 S1	14.509
	ST(d18:0+pO/25:0)	923.6732	C49 H97 O12 N1 S1	15.87
	ST(d18:1/20:3)	829.5374	C44 H79 O11 N1 S1	14.914
	ST(d18:1/24:0)	891.6469	C48 H93 O11 N1 S1	15.212
	ST(d18:1/24:1)	889.6313	C48 H91 O11 N1 S1	14.521
	ST(d18:1/25:0)	905.6626	C49 H95 O11 N1 S1	15.215
So	So(d16:0+pO)	289.2617	C16 H35 O3 N1	2.184
TG	TG(16:0/16:0/18:1)	832.752	C53 H100 O6	13.365
	TG(16:0/16:0/18:1)	832.752	C53 H100 O6	14.404
	TG(16:0/17:0/18:1)	846.7676	C54 H102 O6	15.523
	TG(16:0/18:1/18:1)	858.7676	C55 H102 O6	14.463
	TG(18:0/16:0/16:0)	834.7676	C53 H102 O6	14.898
	TG(18:0/16:0/18:1)	860.7833	C55 H104 O6	15.29
	TG(18:0/18:0/18:1)	888.8146	C57 H108 O6	14.168
	TG(18:0/18:1/18:1)	886.7989	C57 H106 O6	15.513

	TG(18:0/18:1/20:4)	908.7833	C59 H104 O6	15.354
	TG(18:0/8:0/18:0)	750.6737	C47 H90 O6	13.478
	TG(18:1/18:1/18:1)	884.7833	C57 H104 O6	14.898
	TG(18:1/18:1/18:1)	884.7833	C57 H104 O6	15.461
dMePE	dMePE(16:0p/18:1)	729.5672	C41 H80 O7 N1 P1	34.306
	dMePE(16:1p/18:1)	727.5516	C41 H78 O7 N1 P1	33.462
	dMePE(16:1p/20:1)	755.5829	C43 H82 O7 N1 P1	34.414
	dMePE(16:1p/20:4)	749.5359	C43 H76 O7 N1 P1	32.918
	dMePE(16:1p/22:4)	777.5672	C45 H80 O7 N1 P1	33.747
